# Microdosing Sprint Distribution as an Alternative to Achieve Better Sprint Performance in Field Hockey Players

**DOI:** 10.3390/s23020650

**Published:** 2023-01-06

**Authors:** Víctor Cuadrado-Peñafiel, Adrián Castaño-Zambudio, Luis Manuel Martínez-Aranda, Jorge Miguel González-Hernández, Rafael Martín-Acero, Pedro Jiménez-Reyes

**Affiliations:** 1 Education Faculty, Autonomous University of Madrid, 28049 Madrid, Spain; 2 Center for Sport Studies, Rey Juan Carlos University, 28943 Madrid, Spain; 3 MALab (Movement Analysis Laboratory) Research Group, Faculty of Sport, Catholic University of Murcia (UCAM), 30107 Murcia, Spain; 4 Faculty of Health Sciences, Universidad Europea de Canarias, 38300 La Orotava, Tenerife, Spain; 5 Faculty of Sports Sciences and Physical Education, Universidad de La Coruña, 15179 La Coruña, Galicia, Spain

**Keywords:** microdosing, sprint performance, team sports, field hockey, training load distribution

## Abstract

Introduction: The implementation of optimal sprint training volume is a relevant component of team sport performance. This study aimed to compare the efficiency and effectiveness of two different configurations of within-season training load distribution on sprint performance over 6 weeks. Methods: Twenty male professional FH players participated in the study. Players were conveniently assigned to two groups: the experimental group (MG; *n* = 11; applying the microdosing training methodology) and the control group (TG; *n* = 9; traditional training, with players being selected by the national team). Sprint performance was evaluated through 20 m sprint time (T20) m and horizontal force–velocity profile (HFVP) tests before (Pre) and after (Post) intervention. Both measurements were separated by a period of 6 weeks. The specific sprint training program was performed for each group (for vs. two weekly sessions for MG and TG, respectively) attempting to influence the full spectrum of the F-V relationship. Results: Conditional demands analysis (matches and training sessions) showed no significant differences between the groups during the intervention period (*p* > 0.05). No significant between-group differences were found at Pre or Post for any sprint-related performance (*p* > 0.05). Nevertheless, intra-group analysis revealed significant differences in F0, Pmax, RFmean at 10 m and every achieved time for distances ranging from 5 to 25 m for MG (*p* < 0.05). Such changes in mechanical capabilities and sprint performance were characterized by an increase in stride length and a decrease in stride frequency during the maximal velocity phase (*p* < 0.05). Conclusion: Implementing strategies such as microdosed training load distribution appears to be an effective and efficient alternative for sprint training in team sports such as hockey.

## 1. Introduction

Field hockey (FH) is characterized by its intermittent nature, with alternating periods of high- and low-intensity activity throughout the match [1]. To effectively meet the demands of the game, high-level hockey players must frequently change their speed, with an average frequency of approximately 6.8 s of playing time [2]. As a result, the equivalent distance and intermittent index values are high, indicating significantly higher energy expenditure compared to that required to cover the same distance at a constant speed [1,3]. High-intensity actions such as burst accelerations, top-speed sprinting or changes in direction represent between 17.5 and 30% of the overall activity time [4]. Despite differences between positions [5,6], sprint performance stands in these activities as a differentiating factor between elite and sub-elite FH players [7,8]. According to some authors, several changes in FH could be responsible for this. These include advances in playing materials, the implementation of unlimited substitutions and quick restarts, the addition of a sixth field substitute and the recent restructuring of game formats to four 15 min quarters instead of two 35 min halves (International Hockey Federation, 2019). Such changes, especially at the elite level, have made the sport more demanding overall [6]. Due to the increased competitiveness of high-intensity activities, the importance of enhancing performance in these situations has also increased.

Nevertheless, in field hockey, similar to other sports such as football [7] and rugby [8], the adoption of new training approaches characterized by a greater tactical component and relatively smaller training distances per player compared to actual game play has become prevalent [9,10]. The use of such approaches contributes to the accumulation of a greater number of technical-tactical actions and a greater frequency of high-intensity events. These approaches, commonly referred to as small-sided games, are highly effective at improving cardiovascular fitness and simulating the acceleration and deceleration patterns of official matches. However, the literature suggests that these methods may not be as effective at increasing total volumes of high-speed running or reproducing the most demanding phases of competition [9]. 

The gap between the demands of modern, tactical-based training and high-intensity periods within competitive contexts is a common concern for coaches. To address this issue, coaches may utilize “complementary” sprint training specifically tailored to optimizing maximum speed during these high-demand phases. Complementary training protocols that replicate competitive intensity during training sessions have been shown to enhance physical performance and potentially mitigate the risk of soft-tissue injury [10,11]. However, given the complexity of the elements to be worked on and the specifics of this type of training in terms of intensity and fatigue management, it has been pointedly challenging to introduce them into regular sessions [12,13]. In order to shed some light into these critical contexts, a new approach has emerged in recent years to facilitate the assessment of acceleration mechanical capabilities through analysis of the velocity–time curve obtained during a maximal sprint. This method, known as the horizontal force–velocity profile (HFVP), makes it possible to monitor the capabilities while identifying their specific training needs for sprint performance optimization. Together with all this information, and with the aim of determining a practical way of monitoring neuromuscular fatigue, measuring CMJ height loss has been used as a valid method to provide practical and scientific information about the actual level of fatigue induced by a sprint training session, showing the internal load experimented during a sprint training session, this information being of considerable benefit to coaches for monitoring and as a good internal load marker.

The present study aimed (i) to compare the effect of two different configurations of within-season training load distribution (Microdosing Group (MG) vs. Traditional Group (TG)) on sprint kinetics with a specific focus on the horizontal force–velocity profile (HFVP); and (ii) to compare the fatigue induced by these two approaches as assessed by a daily Countermovement Jump (CMJ) height test [14]. It was hypothesized that the microdosing (MG) group protocol, characterized by a greater workload distribution (i.e., higher frequency of training sessions and a lower volume of loads per session), would exhibit a lower level of fatigue after specific training sessions, potentially leading to varying degrees of adaptation.

## 2. Methods

### 2.1. Participants

Twenty male FH players (age: 24.8 ± 3.9 yrs; height: 1.81 ± 0.05 m; body mass: 78.0 ± 5.0 kg; and BMI: 23.8 ± 1.4) with no history of injury in the previous 6 months participated in the study. The players belonged to a professional Field Hockey Club competing in the First National Division (Spain), and 45% of them were members of the Spanish National Team. All participants had been training in this sport for at least 7 years, and their training load during the study was ~10–12 h of hockey training (5 sessions) plus competitive matches every week. Data collection took place during the regular season, with one competitive match per week. All of the players signed an informed consent form before being enrolled in the study. The participants received information about the study objectives and potential risks associated with the different tests used. The study protocol was approved by the Regional Ethical Review Board and by the Institutional Research Ethics Committee. In addition, the study was conducted following the recommendations of the Declaration of Helsinki.

### 2.2. Study Design

**Figure 1:**
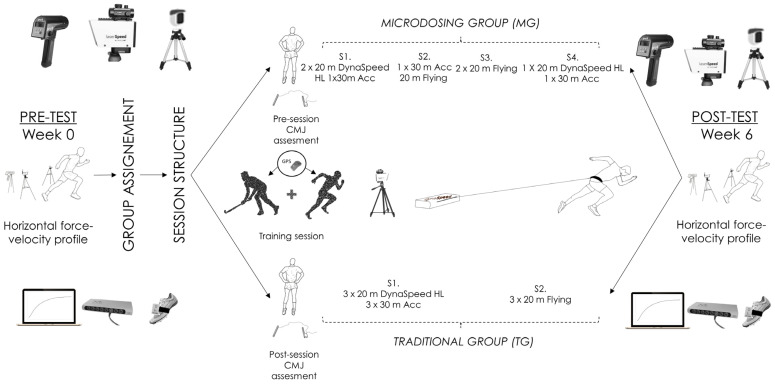
Experimental design and testing structure: T20m: time in 20 m sprint test; HFVP: horizontal force–velocity profile; S: Sessions; CMJ: counter movement jump; Dynaspeed; Acc: accelerations; GPS: global positioning system; W: week; HL: Heavy Load.

A quasi-experimental Pre–Post controlled study was performed (Figure 1), with players assigned to two groups: the microdosing training group (n = 11, 4 weekly training sessions) and the traditional group (n = 9; national team players, 2 weekly training sessions). The main difference between the groups was in the distribution of the specific sprint training loads. This specific training program was developed over a 6-week period. To determine the possible effects of the microdosing training strategy, all players continued with their regular field hockey training sessions (technical–tactical) and were tested both before and after the sprint-training program. The established order was set in order to complete the measurements without interfering with the daily hockey training undertaken on a regular basis. Accordingly, the assessment of sprint performance was evaluated through HFVP tests. Both measurements were separated by a period of 6 weeks, as required for training program implementation. Throughout the intervention, conditional demands for each training session and match were recorded. However, it should be noted that because of their selection by the national team, data for certain sessions of the control group were collected under different training conditions. In turn, jump height losses in the CMJ test were evaluated before and after the sprint training sessions to examine the effects of these on neuromuscular performance. All training sessions were supervised by a strength and conditioning specialist and were performed on an outdoor, synthetic grass FH playing field.

### 2.3. Assessment Protocol

All of the Pre and Post tests were preceded by a standardized warm-up (6 min running at 8–10 km/h with general conditioning exercises and dynamic stretching) and a specific warm-up depending on the test to be performed. Likewise, all tests were performed at the same time of day for each player in order to avoid any negative effects of circadian rhythms. Verbal encouragement was provided by the research staff during all tests.

### 2.4. Equipment and Data Acquisition

The specific training program was developed over a 6-week period. For the training intervention and data collection, the following systems were used to measure the velocity during Pre and Post testing sessions: (1) linear motorized system Dynaspeed (Ergotest Technology AS, Langesund, Norway); (2) laser (Muscle LabTM Laser Speed device Ergotest Innovations, Stathelle, Norway); (3) IMUs (Ergotest Technology AS, Langesund, Norway); (4) radar (Stalker Pro II Sports Radar Gun; Plano, TX, USA); and (5) timing gates (Witty Microgate, Microgate, Bolzano, Italy). For the different training sessions, in order to train and monitor effects, the following systems were used: (1) linear motorized system Dynaspeed (Ergotest Technology AS, Langesund, Norway); (2) GPS (GPS, SPI ELITE, GPSport, Fyshwick, Australia); and (3) opto-electronic timing system for jumping Optojump (Microgate, Bolzano, Italy).

A brief description of the main features o these devices are presented below:

(1) Linear motorized system Dynaspeed (Ergotest Technology AS, Langesund, Norway): This device was used to measure velocity–time curves under different resistance loads imposed by the motorized system. The device was placed on the field, 2 m behind the starting position. The player was connected to the Dynaspeed via a cable attached to a waist belt. Raw velocity data were computed at 1000 Hz from the change in position of the cable and were recorded on the specific software of Musclelab.
(2) Laser (Muscle LabTM Laser Speed device Ergotest Innovations, Stathelle, Norway): The device was set on a tripod on the track, 3 m behind the starting position and 1 m above ground level, corresponding approximately to the height of participants’ center of mass [15]. Laser system calculate velocity measuring the time delay of pulsed infrared light that is reflected off the subject [15]. Raw velocity data were sampled at 1000 Hz, recorded, and smoothed by the software supplied by the manufacturer (Muscle LabTM, version 10.200.90.5097, Stathelle, Norway).
(3) IMUs (Ergotest Technology AS, Langesund, Norway): The combined laser + IMU system (Laser Speed) or Dynaspeed + IMU system, as part of the MUSCLELAB system (Ergotest Technology AS, Langesund, Norway), recorded distance over time continuously during each attempt. Throughout each sprint, contact and flight times together with step length (distance between two adjacent contact times measured with laser) and frequency (1/contact + flight time step) were automatically detected by the software using wireless 9-degrees-of-freedom IMUs integrated with a 3-axis gyroscope attached on top of the shoelaces of the spikes of each foot directly up the IMUs of the 3D-IMU system. The sampling rate of the IMU was 1000 Hz with maximal measuring range of 2000°·s^−1^ ± 3% (Ergotest Technology AS, Langesund, Norway). All recordings of the IMUs and the laser were synchronized with MUSCLELAB v10.57 (Ergotest Technology AS, Langesund, Norway). This system has been previously reported to be a valid system compared with force plates [16], with the results of that study showing that laser + IMU systems are as accurate at measuring step-by-step kinematics as force plate systems.
(4) Radar (Stalker Pro II Sports Radar Gun; Plano, TX, USA): The device was set on a tripod on the track, 5 m behind the starting position and 1 m above ground level [15]. The raw velocity–time curve was measured at a sampling frequency of 46.875 Hz. Then, the cleaned data were fitted using the exponential model proposed and were validated by Samozino and colleagues [17] in order to compute the sprint mechanical outputs.
(5) Timing gates (Witty Microgate, Microgate, Bolzano, Italy): Dual-beam timing gates were placed on the track 1 m above ground level at 0, 10, 20, and 30 m from the starting line to monitor training sessions of free sprinting. The starting position was located 0.5 m behind the first timing gate.
(6) GPS (GPS, SPI ELITE, GPSport, Fyshwick, Australia): The GPS units provided a sampling rate of 10 Hz and encompassed a double constellation system (GNSS and GPS). They were tightly installed into a fitted vest on the upper thoracic spine between the scapulae. Time–motion variables such as distance, meters per minute, high speed running, number of sprints, metabolic power and high-intensity accelerations were measured during the 2 weekly FH training sessions and league matches over the course of the intervention.
(7) Opto-electronic timing system for jumping Optojump (Microgate, Bolzano, Italy): The Optojump photoelectric cells, which consist of two parallel bars (one receiver and one transmitter unit, each measuring 100 × 4 × 3 cm), were placed approximately 1 m apart and parallel to each other. The transmitter contained 32 light emitting diodes, which were positioned 0.3 cm from ground level at 3.125 cm intervals. Optojump bars were connected to a personal computer, and the proprietary software (Optojump software, version 3.01.0001) was used to perform jump height quantification. The Optojump system measured the flight time of vertical jumps with an accuracy of 1/1000 s (1 kHz). Jump height was then estimated as 9.81 × flight time^2^/8 [18].

#### 2.4.1. Anthropometric Measures

Height and body mass were measured prior to the training program implementation using a professional weighbridge (OMRON^TM^- Model BF-511).

#### 2.4.2. CMJ Height Loss Test

Jump height loss was determined by comparing 3 valid attempts with 20 s rest between attempts in pre-fatigue (just after warm-up) vs. post-fatigue situations (immediately after training session). An opto-electronic timing system (Optojump, Microgate, Bolzano, Italy) was used for this purpose. All players had previous experience with the specific jumping technique involved, but special care was taken regarding the landing position (feet and knees). After the standard warm-up, a specific warm-up was carried out based on 2 progressive sets of 5 submaximal–maximal CMJs. During the CMJ, the participant was instructed to rest their hands on their hips while performing a downward movement to 90° of knee flexion followed by a vertical jump of maximum effort. All participants were instructed to keep their knees straight during the flight phase of the jump and to land in an upright position.

#### 2.4.3. Sprint Acceleration Horizontal Force–Velocity Profile (HFVP)

Two maximal sprints separated by a 3 min rest were performed to determine the horizontal force–velocity profile for each player. A warm-up protocol incorporating several sets of progressively faster running accelerations was followed at both Pre and Post. HFVP was derived from running speed–time measurements using a laser device with a sampling frequency of 1000 Hz (Muscle LabTM, version 10.200.90.5097, Stathelle, Norway) according to the methods described in [17,19]. The starting position was set with the front foot located 1 m behind the starting line. Participants were required to give an all-out maximal effort in each sprint, and the fastest trial was kept for further analysis.

#### 2.4.4. Maximal Speed Kinematic Stride Characteristics

Once data collection was completed according to the procedure described above, the most relevant spatiotemporal kinematic variables, such as contact and flight time, stride time, step length and frequency or step velocity during the top-speed phase, were analyzed. The data used for this analysis therefore correspond to the average of the best attempts recorded in the Pre and Post-test assessments. These spatiotemporal kinematic variables were measured using the linear motorized system Dynaspeed, laser, IMUs, and the synchronization of the IMU system with the laser and linear motorized system.

#### 2.4.5. Quantification of Specific Field Performance Variables

Time–motion variables such as distance, meters per minute, high-speed running, number of sprints, metabolic power and high-intensity accelerations were measured during the 2 weekly FH training sessions and league matches over the course of the intervention. For this purpose, a GPS device (GPS, SPI ELITE, GPSport, Fyshwick, Australia) with a sampling rate of 10 Hz was used during the entire 6-week implementation of the training program.

### 2.5. Reliability of Measurements

The test–retest reliability of the 30 m sprint time was assessed using the coefficient of variation (CV) and the intraclass correlation coefficient (ICC) with 95% confidence intervals (95% CI). The results indicated a very high level of reliability (CV: 0.6%, ICC: 0.994 (0.975–0.999)). The average value of the three jumps was used for subsequent statistical analysis, which revealed the countermovement jump test to be reliable, with a CV of 2.7% and an ICC of 0.978 (0.958–0.998). The measurements related to force–velocity profile (FVP), including F0, v0, Pmax, and RF, demonstrated acceptable reliability for all outcomes (CV 1.2–2.9%; ICC 0.93–0.98). Similarly, step kinematic variables such as contact time, flight time, stride time, step length, step frequency, and step velocity also demonstrated acceptable reliability (CV 0.9–2.5%; ICC 0.95–0.99).

### 2.6. Training Protocol

The implementation of a specific sprint training program was carried out in order to influence the whole force–velocity spectrum. To achieve this goal, three different sprint modalities were combined: a 20-m sprint with a heavy load (HL) using the Dynaspeed system, which resulted in a 45–60% decrease in velocity; acceleration sprints over a distance of 30 m; and a 20-m flying sprints. Each of these sprint protocols was implemented in an effort to optimize sprint performance and impact the force–velocity profile. Depending on the group and session, the sprints were distributed as shown in Figure 1. The same training volume was distributed across 4 vs. 2 weekly sessions for MG and TG, respectively. The sprint training sessions took place in the afternoon (5–7 pm) before the field hockey training, over a 6-week period. Each training session consisted of 25–30 min with an 8–10 min standard warm-up consisting of running at 8–10 km/h, dynamic exercises, and a specific warm-up based on short sprints with and without low-load sleds. The research staff provided verbal encouragement during all training sessions performed in the sprint training program.

### 2.7. Statistical Analysis

Data are reported as mean ± standard deviation (SD). Statistical analyses were performed using JASP (JASP Team, 2019; jasp-stats.org). Data distribution for normality and homogeneity of variance across groups was examined using the Shapiro–Wilk and Levene’s test, respectively. A 2 (group) × 2 (time) repeated measures ANOVA was calculated for each parameter. Bonferroni post hoc tests were used when the interaction was significant. Statistical significance was established at the *p* < 0.05 level. Effect sizes (ESs) were calculated using Cohen’s d on the pooled SD. Interpretation of the magnitude of the ES was performed as follows: <0.2, 0.25–0.5, 0.5–1.0, >1.0 for trivial, small, moderate, and large, respectively. Likewise, the difference in height of the CMJ before and after the session, together with the quantification of the training load, were analyzed using independent samples t-test and Cohen’s d.

## 3. Results

No significant differences were found between the groups for any of the parameters examined concerning the conditional demands collected during matches or training sessions during the intervention period. On the other hand, the average jump height loss recorded following sprint-specific training sessions did show significant differences between groups, indicating different levels of neuromuscular fatigue (2.27 ± 0.04 (MG) vs. 4.72 ± 0.11 (TG) cm *p* < 0.001; ES: −0.36 (LL: −4.49; UL: −2.79)) (Table 1).

The effects of both training protocols on the physical qualities and performance parameters evaluated during acceleration, along with mechanical effectiveness, are shown in Table 2. No significant between-group differences were found for any HVFP-related variable at either Pre or Post test. Nevertheless, the intra-group analysis revealed that only MG showed significant differences for the Pre–Post training program in F0 (N/Kg), Pmax (W/Kg), Mean RF on 10 m, T5 (s), T10 (s), T15 (s), T20 (s), T25 (s), Distance in 2 s (m) and Distance in 4 s (m).

**Figure 2:**
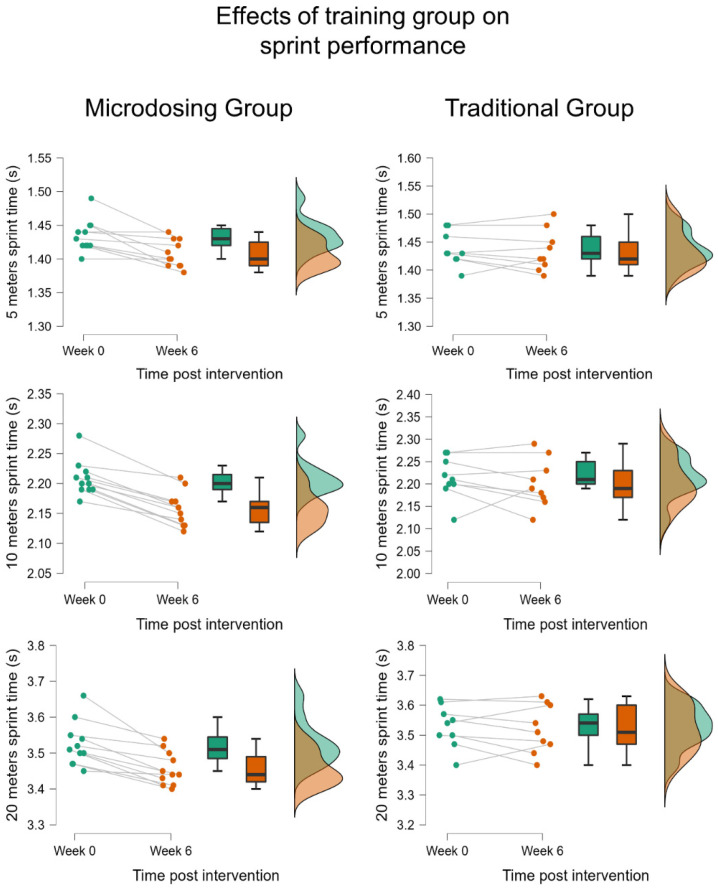
Effects of training group on sprint performance. Colours are indicating the values before (green) and after the training intervention (orange). But it is the same in the left and right columns because the left column is for Microdosing group and right column is for the traditional group.

For a more detailed understanding, the individual responses for some of the most representative performance parameters and HFVP are illustrated in Figure 2.

## 4. Discussion

The present study was designed to explore the effects of two different models of sprint-specific training load distribution on the mechanical outputs of the sprint performance in professional FH players. Attending to the magnitude of the observed intra-group effects, the present study found that the distribution of sprint training volume has a significant impact on performance outcomes, with the microdosing approach being an efficient method for improving acceleration capacity while minimizing fatigue. These findings were evident in the significant improvements observed in most sprint parameters among the microdosing group, though no significant differences were detected between the two groups when compared overall.

As discussed in several studies, the nature of the competitive microcycle in the vast majority of team sports is complex [20,21]. During this period, the allocation of training loads was carefully planned based on important considerations such as the recovery time required for specific elements and the time leading up to competition [22]. Given the high number of elements that must be addressed throughout the training microcycle, the subordination of the design and distribution of training loads based on the distance between competitions or the prioritization of integrated tasks over more analytical ones creates a scenario in which the preparation of the more conditional components is deemed secondary, entailing important implications for both performance and injuries [23]. Recent publications have shown that tactically oriented approaches do not represent an effective stimulus when the objective is to prepare players for the most demanding phases of the game, where sprinting plays a fundamental role [24].

This study endeavored to examine approaches that, while frequently utilized in sports, have yet to be empirically validated for their effectiveness. The analysis of the results showed that, although both groups received the same volume of training (either hockey or sprint specific), only those who used a microdosing approach achieved significant intra-group improvements in their 5, 10, 15, 20, and 25 m times (−0.03 s, −0.05 s, −0.06 s, −0.07 s, −0.09 s, respectively). The magnitude of the changes reported for the microdosing group on sprint performance variables is slightly greater than those observed in the literature [25]. In contrast to previous reports, the effects of training in the traditional sprint group were not statistically significant; however, the volume of weekly training and the target population should be noted as factors that could influence this adaptation. While training volume compared to other combined interventions [26,27,28,29] falls in similar ranges (~200–300 m per week), the populations examined display markedly different competitive standards and, in most studies, focusing on team sports, weekly training loads are not reported. Despite the dearth of literature utilizing a similar microdosing approach, it is interesting to compare the present findings with studies examining the impact of various training load distributions on strength adaptations. Ochi et al. [30] reported the superiority of protocols with a higher training frequency (versus equal volumes of traditional resistance training) with respect to adaptation-related variables in strength training. Their findings are not only in line with those shown above in terms of performance improvements (in their case in strength), they also report a higher RPE for those groups with a lower load distribution, therefore providing supporting evidence that lower degrees of fatigue may lead to a higher degree of adaptation. Nevertheless, it is worth noting that while these authors did find significant between-group differences for maximal voluntary contraction (MVC) at week 11, we only found such differences for intra-group factors (i.e., Pre–Post) in MG. Similarly, cluster-based resistance training was used to explore various loading configurations capable of optimizing the stimulus provided to athletes [31]. These protocols revealed that effort distribution plays a key role in the adaptations generated under volume-matched training contexts [32]. In this sense, resistance training through the cluster configuration would share with microdosing strategies the pursuit of greater adaptation by limiting fatigue and performing each repetition at a higher intensity compared to traditional load distributions. Recently, Pareja-Blanco et al. [33] and Jiménez-Reyes et al. [34] conducted studies on the relationship between the level of fatigue experienced by athletes during a session (measured, among other ways, by changes in jump height) and their adaptive responses. These authors demonstrated that a lower degree of fatigue during strength training not only leads to greater strength gains, it also translates to improved performance in skills such as jumping or sprinting without including these specific skills in the training program. Adapting these kinds of approaches to sprint training may potentially solve problems related to training volume accumulation while minimizing negative interference with training dynamics [7,11,24,35]. Regarding adaptations, for athletes, coaches and scientists, it can be of great importance to know the step-by-step kinematics to check how they respond to a stimulus (after a training intervention). In our study, we observed that the use of a microdose-based approach to distributing workload may have resulted in enhanced training efficiency. This finding may be attributed to the higher level of stimulus specificity in the microdosing group. Specifically, the microdosing group experienced lower levels of fatigue during each training session, despite having a similar overall weekly training volume. The distribution of fatigue across the sessions was more evenly controlled in the microdosing group, potentially enabling a more targeted and effective stimulus to be applied. In our case, the use of the laser-IMU system, which been proved to be a valid system on the basis of a comparison with force plates [16], was a good opportunity to check the evolution in these parameters. The Dynaspeed system allowed for session-by-session monitoring of these parameters, and, when combined with the laser-IMU system, facilitated the implementation of step-by-step kinematics training with direct feedback to athletes. Our findings suggest that the significant increases in step velocity (~8.25 m/s to ~8.41 m/s) observed in the top speed phase for the MG group were likely due to an enhancement in step length (~1.84 m to ~1.97 m) coupled with a reduction in step frequency (~4.51 steps per second to ~4.27 steps per second).

Although no significant differences between groups were found at Post, the microdosing group exhibited a greater intragroup (Pre–Post) magnitude of effects, which was accompanied by a significantly smaller reduction in CMJ height recorded across the various training sessions. This difference in the magnitude of the changes may be partially explained by studies proving the superiority of training sessions in which the training intensity remains elevated throughout the session as a result of a reduced workload [36]. The impact of these intensity losses has been repeatedly contrasted in the scientific literature and is linked not only to mechanical fatigue indices (jump height, movement velocity or sprint performance decrements), but also to biochemical [37] and hormonal markers [38,39]. As can be inferred from recent evidence [36,40], the interrelation of all of these factors creates an environment that, depending on its configuration, may modify the training stimulus received, facilitating or limiting certain adaptations. Accordingly, a microdose-based load distribution appears to ensure greater training efficiency by guaranteeing a higher specificity of the stimulus received, because of the lower distortion generated by current fatigue. This, together with in-season training-oriented protocols, would provide optimal stimuli for improving performance without compromising neuromuscular performance in well-trained athletes [41].

The results of the study showed that there were only intra-group (Pre–Post) changes in the mechanical components of acceleration for the MG group, but no differences between groups were observed after both groups followed the same overall training program. Specific sprint training loads were implemented with the goal of stimulating the entire force–velocity spectrum, taking into account the crucial role of improved sprint abilities as determinant actions for elite field hockey [8]. Nevertheless, only MG showed significant changes in F0 and Pmax after the intervention (Table 2). Although these adaptations led to statistically significant decreases for each intermediate split time measured (t5–t25), training oriented towards the simultaneous improvement of all sections of the force–velocity spectrum only produced significant improvements for those phases where force was applied at very low speeds. The findings found in this study are in line with those reported by Mendiguchia et al. (2020) [26], where F0 and Pmax are mainly responsible for the improvement of sprint performance over 5 and 20 m. The training program designed in this paper was intended to develop the entire force–velocity spectrum in a uniform manner. However, given the complex requirements involved in the production of force at high speed, sustained high-intensity training sessions may not be enough to produce adaptations. The incorporation of additional elements affecting the coordinative skills linked to increased performance throughout these phases should be explored, as noted recently by other authors [26,29].

### Limitations

While promising, the effect of the type of training load distribution across specific sprint training programs needs to be explored in more detail. Despite being complex to implement in high-performance contexts, future work along these lines should seek to implement randomized rather than convenience assignments in order to rule out possible biases associated with the training population and groups themselves.

## 5. Practical Applications

This study was the first to explore the effect of microdosing of sprints during training sessions. The results demonstrated the ease of implementation of both protocols (especially the MG approach) to real workload dynamics in a high-level team. Aspects such as the frequency of training, the time spent for each specific training, or the degree of fatigue allowed play as important a role as elements more frequently discussed in the literature, such as the selection of exercises, and the intensity or the volume of training. In order to optimize sprint performance, it is essential for coaches and athletes to carefully consider their training workload distribution strategy. Confirmation of the validity of this approach offers practitioners new alternatives for implementing an effective and time-efficient stimulus to optimize sprint capabilities. These results may not only have important implications for acceleration performance, they may also be helpful during the management of injury processes.

## 6. Conclusions

The present study found that the distribution of sprint training volume significantly affects performance outcomes in professional field hockey players, with the microdosing approach being effective in improving acceleration capacity while minimizing fatigue. The traditional sprint group did not show significant performance improvements, although training volume and the target population may have influenced this outcome. These findings align with previous research showing the superiority of protocols with a higher training frequency in strength training, leading to higher degrees of adaptation due to lower degrees of fatigue. Further research is needed to validate the effectiveness of the microdosing approach in sports and to determine the optimal training load distribution for various sports and performance outcomes.

## Figures and Tables

**Table 1:** Descriptive data of quantified performance variables in matches and training sessions in Field Hockey.

Training Load	Microdosing Group		Traditional Group	
	Training Session	Match	Training Session	Match
Distance (m)	6696.5 ± 745.2	8718.9 ± 1055.06	6862.5 ± 716.6	8875.7 ± 1186.79
Meters per minute (m/min)	60.07 ± 6.7	105.05 ± 12.7	61.6 ± 6.5	106.94 ± 14.3
High Speed Running (HSR) (m)	545.05 ± 113.4	811.66 ± 201.4	557.77 ± 125.7	889.14 ± 175.2
Sprint Running Distance (SPR) (m)	87.6 ± 31.3	138.24 ± 38.8	87.83 ± 33.2	144.75 ± 32.6
Metabolic Power (Pmet) (W·Kg^−1^)	5.46 ± 0.5	6.26 ± 0.8	5.62 ± 0.6	6.41 ± 0.9
High Intensity Accelerations (n)	15.41 ± 4.5	12.39 ± 2.8	16.44 ± 4.9	13.08 ± 3
**Neuromuscular Fatigue**				
CMJ height loss (%)	2.3 ± 0.24 †	-	4.7 ± 0.36	-

Data are presented as mean ± SD. HSR: Meters traveled at >19 km/h. SPR: Meters traveled at >23 km/h. †: Statistically significant between-group differences.

**Table 2:** Differences in Pre–Post data for microdosing (MG) and traditional (TG) training groups for physical qualities evaluated during the acceleration ^1^, mechanical effectiveness ^2^, performance parameters during the acceleration ^3^ and maximal speed kinematic stride characteristics ^4^.

		Microdosing Group			Traditional Group		
		PRE	POST	Δ%; ES 95%CI (LL; UL)	PRE	POST	Δ%; ES 95%CI (LL; UL)
**HFVP & Sprint Perfomance**	F0 (N/Kg) ^1^	6.66 ± 0.31	6.95 ± 0.30	**4.35; 0.82 (0.11; 0.82)**	6.83 ± 0.40	6.83 ± 0.45	0.04; 0.01 (−0.65; 0.66)
	V0 (m/s) ^1^	8.79 ± 0.27	8.94 ± 0.27	1.71; 0.55 (−0.18; 0.55)	8.79 ± 0.26	8.84 ± 0.3	0.57; 0.15 (−0.6; 0.91)
	Pmax (w(Kg) ^1^	14.6 ± 0.73	15.5 ± 0.62	**6.16; 1.00 (0.24; 1.77)**	15.02 ± 1.04	15.10 ± 1.22	0.67; 0.09 (−0.57; 0.74)
	Mean RF on 10 m ^2^	0.30 ± 0.01	0.31 ± 0.01	**2.80; 0.80 (0.04; 1.56)**	0.30 ± 0.01	0.30 ± 0.01	1.32; 0.40 (−0.35; 1.14)
	Radar Top Speed (m/s) ^3^	8.24 ± 0.20	8.41 ± 0.23	2.06; 0.73 (−0.09; 1.55)	8.22 ± 0.22	8.23 ± 0.26	0.12; 0.06 (−0.76; 0.88)
	Laser Top Speed (m/s) ^3^	8.23 ± 0.22	8.40 ± 0.26	2.06; 0.71 (−0.11; 1.52)	8.25 ± 0.22	8.25 ± 0.24	0.12; 0.01 (−0.81; 0.82)
	T5 (s) ^3^	1.44 ± 0.02	1.41 ± 0.02	**−2.08; −0.98 (−1.79; −0.18)**	1.44 ± 0.03	1.43 ± 0.04	−0.69; −0.12 (−0.84; 0.60)
	T10 (s) ^3^	2.21 ± 0.03	2.16 ± 0.03	**−2.26; −1.25 (−2.16; −0.35)**	2.21 ± 0.05	2.20 ± 0.05	−0.45; −0.31 (−1.06; 0.45)
	T15 (s) ^3^	2.89 ± 0.05	2.83 ± 0.04	**−2.00; −1.75 (−2.69; −0.77)**	2.89 ± 0.05	2.88 ± 0.07	−0.23; −0.10 (−0.75; 0.56)
	T20 (s) ^3^	3.53 ± 0.06	3.46 ± 0.05	**−1.98; −1.05 (−1.84; −0.27)**	3.53 ± 0.07	3.52 ± 0.08	−0.28; −0.14(−0.80; 0.52)
	T25 (s) ^3^	4.15 ± 0.07	4.06 ± 0.05	**−1.97; −2.20 (−3.30; −1.06)**	4.15 ± 0.08	4.13 ± 0.09	−0.59; −0.39 (−1.06; 0.30)
	Distance in 2 s * (m) ^3^	8.45 ± 0.23	8.71 ± 0.18	**3.00; 1.57 (0.66; 2.46)**	8.42 ± 0.27	8.45 ± 0.34	0.36; 0.14 (−0.52; 0.79)
	Distance in 4 s * (m) ^3^	23.63 ± 0.54	24.32 ± 0.49	**2.85; 1.93 (0.90; 2.94)**	23.75 ± 0.62	23.96 ± 0.70	0.44; 0.17 (−0.59; 0.91)
**Step Kinematics**	Contact time (s) ^4^	0.110 ± 0.01	0.105 ± 0.01	−4.55; −0.63 (−1.34; 0.07)	0.111 ± 0.00	0.110 ± 0.01	−0.90; −0.10 (−0.80; 0.60)
	Flight time (s) ^4^	0.116 ± 0.01	0.129 ± 0.01	**11.21; 0.55 (0.30; 2.32)**	0.116 ± 0.01	0.119 ± 0.08	2.59; 0.26 (−0.62; 1.15)
	Stride time (s) ^4^	0.223 ± 0.02	0.234 ± 0.01	**4.93; 0.97 (0.01; 1.94)**	0.227 ± 0.01	0.229 ± 0.01	0.88; 0.16 (−0.78; 1.10)
	Step length (m) ^4^	1.84 ± 0.12	1.97 ± 0.08	**7.07; 1.54 (0.40; 2.70)**	1.86 ± 0.06	1.88 ± 0.07	1.08; 0.26 (−0.73; 1.24)
	Step frequency (hz) ^4^	4.51 ± 0.32	4.27 ± 0.20	**−5.32; −1.00 (−1.97; −0.02)**	4.42 ± 0.20	4.38 ± 0.18	−0.90; −0.16 (−1.11; 0.78)
	Step velocity (m/s) ^4^	8.25 ± 0.15	8.41 ± 0.18	**1.94; 0.92 (0.12; 1.72)**	8.22 ± 0.17	8.25 ± 0.21	0.36; 0.17 (−0.57; 0.46)

Data are presented as mean ± SD. Bold denotes intra-group differences (*p* < 0.05). ∆%: Percentual differences. Increments or decrements between Pre and Post values (%). * Distance covered during the first 2 or 4 s of acceleration. Data recorded with laser.

## Data Availability

The data presented in this study are available on request from the corresponding author.
